# Precision cancer medicine 2025: some concerns

**DOI:** 10.2340/1651-226X.2025.44604

**Published:** 2025-09-11

**Authors:** Peter Nygren

**Affiliations:** Department of Immunology, Genetics and Pathology, Rudbecklaboratoriet, Uppsala University, Uppsala, Sweden

In the evolving landscape of oncology the concept of ‘precision cancer medicine’ (PCM) stands as one of the most promising frontiers. By tailoring treatment to the unique genetic and molecular profile of each patient’s tumor, PCM offers a vision of cancer treatment that is more effective, less toxic, and personalized [1].

However, the reality is that at present only a minority of patients currently benefit from genomics-guided PCM [2]. Many tumors lack actionable mutations and even when targets are identified, inherent or acquired treatment resistance is often observed. Thus, some concerns about PCM in its current state deserve discussion.

One is the simple issue of the concepts used and that may impact on our way of thinking. PCM as a concept was established approximately a decade ago but one should bear in mind the fact that modern oncology, starting with the introduction of cytotoxic drugs in the 1950s, actually already applied a kind of ‘precision’ from the beginning. Based on preclinical research and clinical observations, drug effects were found associated to and thus guided by, for example cancer diagnosis, disease stage, patient performance status, and comorbidity. Such general and simple ‘biomarkers’ formed a basis for an early version of PCM, although most oncologists would rather denote this ‘empirical cancer medicine’.

The rapidly expanded knowledge in cancer biology and access to large scale methods for genomic analysis have now made it possible to establish a great number of drug–genomic biomarker relationships that open for more advanced PCM. Thus, molecular characterization of the patient’s tumor sometimes allow us to abstain a treatment that will not work or chose a treatment that, on a group level and to various degrees, will increase the probability of benefit [3]. This, of course, is progress but hardly deserves the concept ‘precision’. PCM at its present stage is rather suggested to be regarded and conceptualized as ‘stratified cancer medicine’.

Another concern relates to the fact that PCM is currently strongly focused on genomics with much less interest to investigate and apply other biomarkers to guide cancer treatment for improved efficacy. Although large-scale analysis of the tumor genome as a basis for treatment selection is promising indeed, it should be reminded that there are a number of complex layers of biology that attenuate or even completely remove the impact of genomic changes on outcome at the tissue and organism levels [3, 4], illustrated, for example by the different predictive impacts of BRAF mutations depending on tumor type [3]. However, an excellent exception is illustrated by the impressive efficacy of tropomyosin receptor kinase (TRK) inhibitors in neutrophic receptor tyrosine kinase (NTRK) fusion-positive tumors that opens for truly diagnosis agnostic therapy based on genomics alone [5].

Related and also problematic is the fact that current PCM is often used interchangeably with ‘personalized cancer medicine’. This is even further away from reality than PCM since a personalized approach reasonably means a truly tailored treatment based on the predictive power from a joint analysis of all biomarkers possible, not only genomics, and selected from all drugs available, also including those not currently labelled cancer drugs but that may still modify cancer behavior [6, 7]. Such ‘personalized cancer medicine’ is indeed a long-term goal but use of this concept at the present stage is confusing.

Additional layers of biomarkers need to be added to move the PCM concept forward. Examples are biomarkers reflecting pharmacokinetics, including therapeutic drug monitoring, and pharmacogenomics, that should be applied and developed further to allow for individualized and optimal drug dosing [8]. Other layers of treatment predictors in need of consideration are other ‘omics’ biomarkers, imaging, histopathology, patient nutrition, comorbidity, and use of concomitant drugs including antibiotics that may impact the gut microbiome and subsequently the effect of cancer drugs [6, 9, 10]. Only when bringing information from many such biomarkers into a complex, reasonably AI-generated, treatment predictor this will allow for true PCM and its advancement into personalized cancer medicine. Principles for such approach have been outlined and should now form the basis for clinical trials [11, 12].

Yet another concern in the genomics based PCM discussion is the need to distinguish between its application in routine healthcare and its further development based on research. The former is in principle very simple; it is the routine use of analyses of specific genomic biomarkers proven on the basis of controlled clinical trials to provide benefit in terms of established hard endpoints such as overall survival and quality of life/symptom improvement. A recent example is the addition of encorafenib and cetuximab to the FOLFOX regimen in the first line treatment of metastatic colorectal cancer in the case of a BRAF-mutation [13]. Another is use of TRK inhibitors in NTRK fusion-positive tumors as mentioned above. Such genomics based PCM is not controversial but is mainly a matter of implementation and equity in access.

Genomics-based tumor agnostic PCM in the absence of clinical evidence showing benefit, on the other hand, is a complicated research matter. A number of clinical trials investigating this concept have been performed and reported, mostly with essentially identical design; patients with advanced cancer refractory to standard guideline directed therapy have been recruited, their tumor analyzed for the presence of genomic changes possible to target with a drug and if a target is present and the drug available, patients start treatment followed by the early assessment of drug efficacy [2, 14].

Typical reporting from these trials usually includes endpoints rather to be regarded as surrogates for true benefit, for example the fraction of patients with a successful genomic analysis, a targetable finding, starting targeted drug treatment, and outcome in terms of tumor response and disease control rate at a specific time point. In light of the considerable attrition in number of patients in each step of these trials, the considerable heterogeneity between patients in terms of cancer type, prognostic features and without a control group, it is difficult to draw conclusions on true clinical benefit from the approach. This may also apply for the genomics-based tumor agnostic treatments approved based on such trials [3, 4].

Yet, the mere technical access to this approach combined with seemingly promising findings from these types of trials have made not only cancer patients, their relatives, and media but also healthcare professionals to conclude that this simple tumor agnostic approach is an appropriate way to identify a treatment with great prospects to work. This notion is difficult to handle in relation to patients within routine cancer care and may lead to the ordering of costly genomic analyses and treatment attempts beyond what is considered standard but not part of research [15, 16].

It is acknowledged that the tumor agnostic studies have been necessary to perform to test the overall feasibility of the approach but given the many trials using this design already reported and proving technical feasibility, it is now time to move to study designs that allow for more definitive conclusions on the clinical benefit from use of genomics guided treatment selection, not the least in light of lack of benefit in randomized clinical trials [17–19].

Although it is acknowledged that the phase III controlled randomized design considered standard for proving drug efficacy might be difficult to apply in full in this field, some kind of sufficiently strong comparators need to be included to allow for conclusions applicable in healthcare [4, 14]. Furthermore, studies may be more successful if patents are recruited more selectively based on comprehensive tumor biology knowledge, in earlier lines of treatment applying a ‘window of opportunity’ clinical trial approach and treating patients with drug combinations targeting as many genomic aberrations as possible rather than with single drugs only [20].

In this light it is of concern that many European national clinical trial initiatives ongoing or about to start are still not designed to convincingly show more than feasibility and report mainly surrogate efficacy endpoints. Thus, they recruit a mix of treatment refractory patients and apply a tumor agnostic one gene – one drug approach [21]. Although in the end it will be possible to pool data from these trials to allow for identification of promising combinations of genomic changes and drugs in specific tumor types, definitive conclusions on true benefit will be difficult to draw due to lack of study controls [22]. Comparisons with ‘synthetic controls’ [21, 23] and real world data massively collected within a ‘Digital Oncology Network for Europe’ [24], may be ways forward. However, this does not remove bias or is not yet in place, respectively, ending up with the conclusion that some kind of, preferably simplified, randomized trial design [25] is seemingly necessary to prove benefit [22].

Notwithstanding the above concerns, PCM is undoubtedly a key to substantial progress in cancer treatment. The concerns should instead be seen as an appeal to temper communication about the progress achieved so far, to expand the concept beyond genomics, and to develop PCM within research designed to allow clearer conclusions about patient benefit, and subsequently to identify which parts of the concept are mature enough for cost-effective implementation in routine healthcare.

From a wider perspective, PCM may face barriers to adoption beyond trials. Progress and adoption require coordinated action in evidence, regulation, and equity. Robust data must define where PCM adds most value to ensure clinical benefit and cost-efficiency. Regulatory and reimbursement models should adapt, recognizing real-world data and registry-based evidence alongside trials. Equity is crucial, PCM should not be limited to trial participants or wealthy regions. Shared infrastructures for biomarker analyses and drug access at national/EU levels can expand availability. With scientific rigor and pragmatic health system solutions, PCM can become standard care for all eligible patients.

## References

[1] Mateo J, Steuten L, Aftimos P, André F, Davies M, Garralda E, et al. Delivering precision oncology to patients with cancer. Nat Med. 2022;28:658–665. 10.1038/s41591-022-01717-2 35440717

[2] Song I-W, Vo HH, Chen Y-S, Baysal MA, Kahle M, Johnson A, et al. Precision oncology: evolving clinical trials across tumor types. Cancers. 2023;15:1967. 10.3390/cancers15071967 37046628 PMC10093499

[3] Shah NM, Meric-Bernstam F. The present and future of precision oncology and tumor-agnostic therapeutic approaches. Oncologist. 2025;30:oyaf152. 10.1093/oncolo/oyaf152 40536268 PMC12204399

[4] Westphalen CB, Martins-Branco D, Beal JR, Cardone C, Coleman N, Schram AM, et al. The ESMO tumour-agnostic classifier and screener (ETAC-S): a tool for assessing tumour-agnostic potential of molecularly guided therapies and for steering drug development. Ann Oncol. 2024;35:936–953. 10.1016/j.annonc.2024.07.730 39187421

[5] Cocco E, Scaltriti M, Drilon A. NTRK fusion-positive cancers and TRK inhibitor therapy. Nat Rev Clin Oncol. 2018;15:1–17. 10.1038/s41571-018-0113-0 PMC6419506 30333516

[6] Andrei P, Battuello P, Grasso G, Rovera E, Tesio N, Bardelli A. Integrated approaches for precision oncology in colorectal cancer: the more you know, the better. Semin Cancer Biol. 2022;84:199–213. 10.1016/j.semcancer.2021.04.007 33848627

[7] Armando RG, Gómez DLM, Gomez DE. New drugs are not enough –– drug repositioning in oncology: an update. Int J Oncol. 2020;56:1–34. 10.3892/ijo.2020.4966 PMC7010222 32124955

[8] Groenland SL, van Eerden RAG, Westerdijk K, Meertens M, Koolen SLW, Moes DJAR, et al. Therapeutic drug monitoring based precision dosing of oral targeted therapies in oncology: a prospective multicentre study. Ann Oncol. 2022;33(10):1071–82. 10.1016/j.annonc.2022.06.010 35777707

[9] Blake SJ, Wolf Y, Boursi B, Lynn DJ. Role of the microbiota in response to and recovery from cancer therapy. Nat Rev Immunol. 2024;24:308–325. 10.1038/s41577-023-00951-0 37932511

[10] Passaro A, Bakir MA, Hamilton EG, Diehn M, André F, Roy-Chowdhuri S, et al. Cancer biomarkers: emerging trends and clinical implications for personalized treatment. Cell. 2024;187:1617–35. 10.1016/j.cell.2024.02.041 38552610 PMC7616034

[11] Chang T-G, Park S, Schäffer AA, Jiang P, Ruppin E. Hallmarks of artificial intelligence contributions to precision oncology. Nat Cancer. 2025;6:417–431. 10.1038/s43018-025-00917-2 40055572 PMC11957836

[12] Sadée C, Testa S, Barba T, Hartmann K, Schuessler M, Thieme A, et al. Medical digital twins: enabling precision medicine and medical artificial intelligence. Lancet Digit Health. 2025;7(7):100864. 10.1016/j.landig.2025.02.004 40518342 PMC12412312

[13] Elez E, Yoshino T, Shen L, Lonardi S, Cutsem EV, Eng C, et al. Encorafenib, cetuximab, and mFOLFOX6 in BRAF-mutated colorectal cancer. N Engl J Med. 2025;392:2425–37. 10.1056/NEJMoa2501912 40444708 PMC12197837

[14] Tourneau CL, André F, Helland Å, Mileshkin L, Minnaard W, Schiel A, et al. Modified study designs to expand treatment options in personalised oncology: a multistakeholder view. Eur J Cancer. 2023;194:113278. 10.1016/j.ejca.2023.113278 37820553

[15] Grauman Å, Ancillotti M, Veldwijk J, Mascalzoni D. Precision cancer medicine and the doctor-patient relationship: a systematic review and narrative synthesis. BMC Med Inform Decis Mak. 2023;23:286. 10.1186/s12911-023-02395-x 38098034 PMC10722840

[16] DeCarli K, Bradbury A, Lopez AM, Camacho P, Chatwal MS, Friese CR, et al. Ethical and clinical considerations in ordering and responding to molecular diagnostics and circulating tumor DNA as the science evolves. JCO Oncol Pr. 2024;20:1508–14. 10.1200/OP-24-00481 39531847

[17] Schneider BP, Jiang G, Ballinger TJ, Shen F, Chitambar C, Nanda R, et al. BRE12-158: a postneoadjuvant, randomized phase II Trial of personalized therapy versus treatment of physician’s choice for patients with residual triple-negative breast cancer. J Clin Oncol. 2022;40:345–55. 10.1200/JCO.21.01657 34910554

[18] Andre F, Filleron T, Kamal M, Mosele F, Arnedos M, Dalenc F, et al. Genomics to select treatment for patients with metastatic breast cancer. Nature. 2022;610:343–8. 10.1038/s41586-022-05068-3 36071165

[19] Tourneau CL, Delord J-P, Gonçalves A, Gavoille C, Dubot C, Isambert N, et al. Molecularly targeted therapy based on tumour molecular profiling versus conventional therapy for advanced cancer (SHIVA): a multicentre, open-label, proof-of-concept, randomised, controlled phase 2 trial. Lancet Oncol. 2015;16:1324–34. 10.1016/S1470-2045(15)00188-6 26342236

[20] Fountzilas E, Tsimberidou AM, Vo HH, Kurzrock R. Clinical trial design in the era of precision medicine. Genome Med. 2022;14:101. 10.1186/s13073-022-01102-1 36045401 PMC9428375

[21] Taskén K, Mohammad SFH, Fagereng GL, Falk RS, Helland Å, van Wallwijk van Doorn-Khosrovani SB, et al. PCM4EU and PRIME-ROSE: collaboration for implementation of precision cancer medicine in Europe. Acta Oncol. 2024;63:34791. 10.2340/1651-226X.2024.34791 38779910 PMC11332530

[22] Taskén K, Mahon P. Accelerating precision oncology by converging pragmatic trials and real-world evidence. Nat Rev Drug Discov. 2025; Mar 13, online ahead of print. 10.1038/d41573-025-00047-5 40082591

[23] Popat S, Liu SV, Scheuer N, Hsu GG, Lockhart A, Ramagopalan SV, et al. Addressing challenges with real-world synthetic control arms to demonstrate the comparative effectiveness of pralsetinib in non-small cell lung cancer. Nat Commun. 2022;13:3500. 10.1038/s41467-022-30908-1 35715405 PMC9205915

[24] Mahon P, Chatzitheofilou I, Dekker A, Fernández X, Hall G, Helland A, et al. A federated learning system for precision oncology in Europe: DigiONE. Nat Med. 2024;30:334–7. 10.1038/s41591-023-02715-8 38195748

[25] Kessels R, May AM, Koopman M, Roes KCB. The trial within cohorts (TwiCs) study design in oncology: experience and methodological reflections. BMC Med Res Methodol. 2023;23:117. 10.1186/s12874-023-01941-5 37179306 PMC10183126

